# The curious case of genome packaging and assembly in RNA viruses infecting plants

**DOI:** 10.3389/fgene.2023.1198647

**Published:** 2023-06-08

**Authors:** Tushar Ranjan, Ravi Ranjan Kumar, Mohammad Ansar, Jitesh Kumar, Auroshikha Mohanty, Anamika Kumari, Khushbu Jain, Kumari Rajani, Sailabala Dei, Mohammad Feza Ahmad

**Affiliations:** ^1^ Department of Molecular Biology and Genetic Engineering, Bihar Agricultural University, Bhagalpur, Bihar, India; ^2^ Department of Plant Pathology, Bihar Agricultural University, Bhagalpur, Bihar, India; ^3^ Department of Seed Science and Technology, Bihar Agricultural University, Bhagalpur, Bihar, India; ^4^ Deputy Director Research, Bihar Agricultural University, Bhagalpur, Bihar, India; ^5^ Department of Horticulture, Bihar Agricultural University, Bhagalpur, Bihar, India

**Keywords:** viral genome packaging, energy-dependent, capsid protein, ATPase fold, virus assembly, RNA virus

## Abstract

Genome packaging is the crucial step for maturation of plant viruses containing an RNA genome. Viruses exhibit a remarkable degree of packaging specificity, despite the probability of co-packaging cellular RNAs. Three different types of viral genome packaging systems are reported so far. The recently upgraded type I genome packaging system involves nucleation and encapsidation of RNA genomes in an energy-dependent manner, which have been observed in most of the plant RNA viruses with a smaller genome size, while type II and III packaging systems, majorly discovered in bacteriophages and large eukaryotic DNA viruses, involve genome translocation and packaging inside the prohead in an energy-dependent manner, i.e., utilizing ATP. Although ATP is essential for all three packaging systems, each machinery system employs a unique mode of ATP hydrolysis and genome packaging mechanism. Plant RNA viruses are serious threats to agricultural and horticultural crops and account for huge economic losses. Developing control strategies against plant RNA viruses requires a deep understanding of their genome assembly and packaging mechanism. On the basis of our previous studies and meticulously planned experiments, we have revealed their molecular mechanisms and proposed a hypothetical model for the type I packaging system with an emphasis on smaller plant RNA viruses. Here, in this review, we apprise researchers the technical breakthroughs that have facilitated the dissection of genome packaging and virion assembly processes in plant RNA viruses.

## 1 Introduction

Genome packaging and assembly is a crucial step during the life cycle of RNA viruses infecting plants (Rao, 2006; Ranjan et al., 2021; Kumar et al., 2022; Mohanty et al., 2023). Three different approaches have been reported by which viruses condense their genome inside the empty prohead (Chelikani et al., 2014a; Ranjan et al., 2021; Mohanty et al., 2023). According to the recently upgraded type I classification system present in many of the smaller plant RNA viruses of genome size <20 kb, the processes of assembly and genome packaging involve the coating of the nucleic acid with viral capsid proteins (CPs) in the presence of ATP (Ranjan et al., 2021; Mohanty et al., 2023). On the contrary, a powerful ATP-fueled packaging motor, to package the genome in already assembled proheads of larger viruses with the genome size >20 kb, is involved in both type II and III systems (Burroughs et al., 2007; Chelikani et al., 2014a; Chelikani et al., 20014b). Both type II and III packaging systems have been reasonably well studied so far, but the details of the molecular mechanism of genome packaging, translocation, and assembly processes in plant RNA viruses are rather limited (Ranjan et al., 2021; Mohanty et al., 2023). Therefore, this review aims to update readers on the current knowledge of the molecular mechanism of genome packaging and the steps involved in viral maturation of plant RNA viruses, with a special emphasis on Polerovirus and Potexvirus.

After its entry into a host plant cell, an RNA virus carries out the following steps in a highly coordinated manner to complete a successful infection: 1) uncoating of virions; 2) immediate translation of proteins related to replication; 3) synthesis of new (−) strand and (+) strand RNAs; 4) translation of the movement protein (MP) and capsid protein from newly synthesized viral mRNAs; and finally, 5) the assembly of infectious virions capable of moving from one cell to neighbor cells through plasmodesmata or ultimately interacting with insect vectors for their spreading to healthy plants (Bol, 2005; Catalano, 2005; Rao, 2006; Kumari et al., 2020; Garmann et al., 2022). The entire process of the replication event has been explored in depth in several plant RNA viruses and tremendous reviews have been published worldwide on this subject (Annamalai and Rao, 2005; Bol, 2005; Rao, 2006; Annamalai et al., 2008; Rao et al., 2014). The RNA genome of viruses ranges from monopartite to multipartite, and RNA strands could be of positive or negative senses (Table 1). Table 1 represents the detailed characteristics of RNA viruses infecting plants with suitable examples (Table 1). The plant RNA virus replicates within a cytoplasmic compartment, which is heavily populated by cellular RNAs (Burd and Dreyfuss, 1994; Rao, 2006). Thus, CPs, the RNA-binding proteins, exhibit a remarkable degree of packaging specificity, despite their probability of co-packaging cellular RNAs (Burd and Dreyfuss, 1994; Ranjan et al., 2021; Kumar et al., 2022).

**TABLE 1:** Plant RNA viruses along with their characteristics.

Name of the plant RNA virus	Family	Type of RNA genome	No. of RNA fragments	Host plant
Potato leafroll virus, tobacco necrotic dwarf virus, chickpea stunt disease-associated virus, barley yellow dwarf virus, groundnut rosette assistor virus, and soybean dwarf virus	Solemoviridae	Single-stranded positive-strand RNA	Monopartite	Potato, tobacco, chickpea, barley, groundnut, and soybean
Tomato bushy stunt virus, cucumber soil-borne virus, maize necrotic streak virus, Ahlum waterborne virus, bean mild mosaic virus, *Dianthovirus*, oat chlorotic stunt virus, maize chlorotic mottle virus, and tomato bushy stunt virus	Tombusviridae	Single-stranded positive-strand RNA	Monopartite (except *Dianthovirus*; bipartite)	Tomato, cucumber, maize, and nightshade
Potato virus Y, Blackberry virus Y, longan witches’ broom-associated virus, spartina mottle virus, common reed chlorotic stripe virus, and tobacco etch virus	Potyviridae	Single-stranded positive-strand RNA	Monopartite	Potato, blackberry, longan, chilli, pepper, and tobacco
Tomato spotted wilt orthotospovirus, fig mosaic emaravirus, pigeon pea sterility mosaic emaravirus, raspberry leaf blotch emaravirus, rose rosette emaravirus, and *Pistacia* emaravirus B	Bunyaviridae	Single-stranded negative-strand RNA viruses	Bi- or tripartite	Tomato, fig, pigeon pea, rose, raspberry, onion, and garlic
Beet yellows virus, citrus tristeza virus, carrot yellow leaf virus, wheat yellow leaf virus, strawberry chlorotic fleck-associated virus, and tobacco virus 1	Closteroviridae	Single-stranded positive-strand RNA	Monopartite	Beet, tobacco, citrus, and strawberry
Brome mosaic virus, Semliki Forest virus, alfalfa mosaic virus, olive latent virus-2, cowpea chlorotic mottle virus, and cucumber mosaic virus	Bromoviridae	Single-stranded positive-strand RNA	Tripartite	Bromegrass, tobacco, potato, and cucumber
Potato virus X, *Lolium* latent virus, Cassava virus X, clover yellow mosaic virus, papaya mosaic virus, potato aucuba mosaic virus, and bamboo mosaic virus	Alphaflexiviridae	Single-stranded positive-strand RNA	Monopartite	Potato, papaya, and bamboo
Turnip yellow mosaic virus, grapevine fleck virus, ononis yellow mosaic virus, Cacao yellow mosaic virus, eggplant mosaic virus, and okra mosaic virus	Tymoviridae	Single-stranded positive-strand RNA	Monopartite	Cabbages, cauliflower, broccoli, and eggplant
Tobacco rattle virus, barley stripe mosaic virus, wheat mosaic virus, sorghum chlorotic spot virus, potato mop-top virus, tobacco latent virus, and tomato mosaic virus	Virgaviridae	Single-stranded positive-strand RNA	Mono- or bi- or tripartite	Tobacco, wheat, sorghum, and tomato
Cowpea mosaic virus, cucurbit mild mosaic virus, tomato ringspot virus, tobacco ringspot virus, apple latent spherical virus, and rice tungro spherical virus	Secoviridae	Single-stranded positive-strand RNA	Bipartite	Cowpea, cucurbit, tobacco, tomato, apple, and rice

## 2 CPs with a novel ATPase domain: a new regime in the genome packaging of plant RNA viruses

**FIGURE 1:**
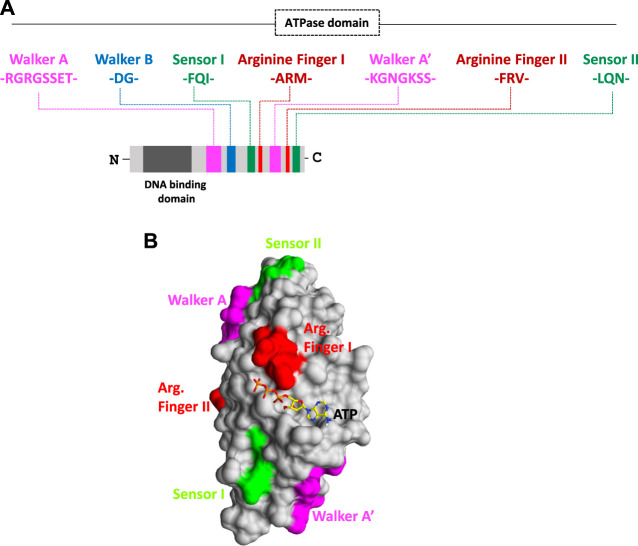
**(A)** Organization of functional motifs on the polypeptide chain of CPs of the Solemoviridae and Alphaflexiviridae family. The functional motifs at the N-terminal ATPase domain are represented in different colors, whereas the C-terminal end occupies the putative DNA-binding domain. Schematics are drawn approximately to the scale and represent the approximate consensus of representative homologs of RNA viruses infecting plants. **(B)** Interaction of the ATP molecule with the I-TASSER-predicted atomic model of CP. All the putative ATP catalysis motifs are highlighted in different colors. For details, refer to Kumar et al., 2022.

In our previous study, an attempt was made to expand and sub-classify type I packaging machinery for several plant RNA viruses, viz., Polerovirus and Potexvirus. These smaller RNA viruses infecting plants fall under an ATP-dependent sub-type IA packaging system, which possess a classical P-loop containing an ATPase domain situated over linear polypeptide chains of CPs (Ranjan et al., 2021; Kumar et al., 2022; Mohanty et al., 2023). The viral encoded CP recognizes the genomic end, nucleates over the viral genome in the presence of ATP, and ultimately, encapsidates them to a mature virion (Ranjan et al., 2021; Mohanty et al., 2023). On the other hand, CPs of viruses belong to type IB, and IC does not possess ATPase folds over it and employs slightly different mechanisms for genome packaging (Ranjan et al., 2021). The type IB virus takes the help of viral- or host-encoded ATPase, whereas the type IC virus prefers an ATP-independent fashion to package their genome inside capsid coats (Ranjan et al., 2021). The role of packaging ATPase in genome packaging is well-known for viruses of type II and III systems, but our recent discovery of the presence of novel ATPase domains on polypeptide chains of CPs has completely changed our perception of genome packaging in plant RNA viruses and raised many interesting facts (Ranjan et al., 2021; Kumar et al., 2022; Mohanty et al., 2023). An ATPase domain comprising different motifs responsible for ATP hydrolysis is situated on the linear primary structure of the CP of almost all plant RNA viruses (Figures 1A, B). Interestingly, apart from RNA viruses, the same ATPase fold consisting of Walker A, Walker B, sensors, and arginine fingers was also found to be present on the polypeptide chain of CPs of DNA viruses infecting plants, such as members of *Nanoviridae* and *Geminiviridae* families. Intriguingly, the presence of very rare patterns of a novel ATPase domain with multiple Walker A, sensor motifs, Walker B, and arginine motifs fetched during our thorough comprehensive analysis has indicated a variation within the ATPase superfamily (Figure 1A) (Ranjan et al., 2021; Kumar et al., 2022; Mohanty et al., 2023). Such variations could be responsible for the evolution of different architectures of an ATPase domain with a single function of ATP hydrolysis during evolution time (Iyer et al., 2004; Chelikani et al., 2014b). These duplicated Walker A, sensors, Walker B, and arginine motifs together form an active site or a pocket for ATP binding and catalysis (Kumar et al., 2022). ATP hydrolysis is coordinated by Walker-A “P-loop” motifs during genome packaging. The Walker A motif with highly conserved sequences of RGRGSSET (lavender color) interacts with β and terminal γ-phosphates of ATP bound at the active site of CPs. On the other hand, the highly conserved Asp residue of Walker B motifs (consensus “hhhhDG”) situated at the tip of the β-strand forms a coordinate bond with the metal ion and further assists in the hydrolysis of ATP (Figures 1A, B) (Kumar et al., 2022). While a conserved Asp residue (blue) coordinates the Mg^2+^ cation, the other conserved Gly residue of Walker-B motifs (blue) involve in a nucleophilic attack toward the terminal γ-phosphate of the ATP molecule bound at active sites by priming a water molecule (Figure 1B) (Ranjan et al., 2021; Kumar et al., 2022; Mohanty et al., 2023).

Intriguingly, all the motifs responsible for binding and catalysis of ATP are either part of the loop or are situated at the top of the β-strand in the generated 3D atomic model of CPs. This is a classical hallmark for the ASCE P-loop ATPase superfamily and makes these critical motifs very flexible for nucleophilic attack and catalysis inside active sites (Figure 1B) (Ranjan et al., 2021; Kumar et al., 2022; Mohanty et al., 2023). The arginine finger I with a consensus sequence “ARM” and the arginine finger II with a consensus sequence “FRV” (in red) are situated at 9–10 amino acid residues next to the sensor I (“FQI” in green) motif and 5–6 amino acid residues downstream of another Walker-A-like motif (represented as Walker A’ in lavender color). Another sensor motif (designated as sensor II with the consensus sequence “LQN” in green color) is present almost after five residues of the arginine finger II. The arginine fingers I and II (red) and sensor motif II (green) are strictly conserved across plant RNA viruses (Figure 1A) (Ranjan et al., 2021; Kumar et al., 2022; Mohanty et al., 2023). The ATPase fold of CPs of plant RNA viruses abides by a unique arrangement of motifs, where arginine fingers I and II and Walker A’ are found to be flanked with sensor I and II motifs from both the sides, and sensor motif I is situated next to Walker B almost after 48 amino acid residues downstream (Figure 1A) (Ranjan et al., 2021; Kumar et al., 2022). This permutation and different combinations of motifs has led to the origin and evolution of different homologs of ATPase folds across the domain of life (Iyer et al., 2004). Atomic structure prediction using I-TASSER revealed that the active site CP comprises all motifs necessary for ATP interaction, except arginine finger I, which is situated far from the rest of the motifs in their folded structure (highlighted in red; Figure 1B). Arginine finger is a classical hallmark of ATPases and is conserved across many ATPases. It completes the active site from a distinct location, forming contacts with the γ-phosphate of the nucleotide (Ranjan et al., 2021; Kumar et al., 2022; Mohanty et al., 2023).

The unique arrangement of ATP-binding motifs in the primary and tertiary structures of CPs indicates the structural similarity of CPs with the members of the well-known classical P-loop ATPase superfamily (Figures 1A, B) (Ranjan et al., 2021; Kumar et al., 2022). The functions of more than one Walker-A-like motifs in ATP binding and catalysis need to be explored. The use of site-directed mutagenesis (SDM) to replace critical amino acid residues in ATPase motifs could provide better insights into understanding the function of these motifs in genome translocation, packaging, assembly, and viral maturation (Ranjan et al., 2021; Kumar et al., 2022; Mohanty et al., 2023). The P-loop was found to play a crucial role in directing ATP binding and hydrolysis with genome packaging, translocation, and assembly in all RNA/DNA viruses (Ranjan et al., 2021; Kumar et al., 2022; Mohanty et al., 2023). Our preliminary experimental data suggest that the recombinant CPs of the potato virus X and potato leafroll virus, overexpressed in the bacterial system, showed enhanced ATP hydrolysis activity in the presence of DNA (T. Ranjan, unpublished data). The replacement of critical amino acid residues of Walker-A and Walker-B motifs obtained using SDM resulted in loss of ATPase function of recombinant CPs, indicating the role of these motifs in ATP binding and hydrolysis (T. Ranjan, unpublished data). Interestingly, we also observed the importance of the Walker A’ motif in ATP hydrolysis, the removal of which led to the loss of ATPase activity of recombinant CPs (T. Ranjan, unpublished data). Thus, our comprehensive sequence analysis and preliminary experimental data indicate the direct role of ATPase folds of CPs in the genome packaging of RNA viruses infecting plants (Kumari et al., 2020; Ranjan et al., 2021; Kumar et al., 2022; Mohanty et al., 2023).

## 3 A model for genome translocation, packaging, and assembly in plant RNA viruses

**FIGURE 2:**
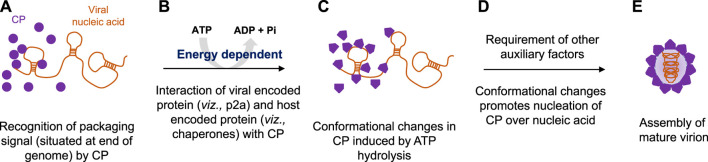
Proposed model for energy-dependent genome packaging and assembly in the plant RNA virus. **(A)** CPs first recognize the packaging signal or pac site, situated at the termini of viral genomes with the help of its RNA-binding domain. **(B)** Replication and genome packaging events in plant viruses are concurrent. Viral-encoded replicase protein (such as p2a in the case of Bromovirus) and sometimes host-encoded proteins (such as chaperonins in the case of Potyvirus) interact with CPs in an energy-dependent manner. **(C)** Binding of ATP molecules to the ATPase domain induces conformational changes in CPs. **(D)** Furthermore, ATP hydrolysis facilitates the nucleation of CPs over viral genomic RNAs, and **(E)** with the help of other auxiliary factors, CP encapsidates the genomic RNA, ultimately leading to the production of mature virion particles.

Plant RNA viruses exhibit a wide range of virion symmetry including rods (e.g., Potyvirus), icosahedral (e.g., Cucumovirus), and bacilliform shape (e.g., alfalfa mosaic virus) (Ford et al., 2013; Rao et al., 2014). Despite these diversities among virus families, mature virions of a particular species often exhibit structural homogeneity and thus share the common mechanism of genome packaging and assembly (Chelikani et al., 2014a; Guo et al., 29019; Larson et al., 2005). Nucleation/oligomerization of capsid proteins around viral RNA genomes and virion assembly involves two primary molecular interactions: 1) protein–protein interactions (viz., capsid–capsid interaction) and ii) RNA–protein interactions (viz., RNA–capsid interaction) (Qu and Morris, 1997; Basnayake et al., 2009; Bunka et al., 2011; Ford et al., 2013). CPs recognize specific packaging signals (sequences) situated usually at the genomic end instead of nucleating at some random sites on a viral genome with the help of the N-terminal DNA-binding domain (Figure 2A) (Garoff et al., 1980; Bink et al., 2003; Zlotnick et al., 2020; Li et al., 2021). Our bioinformatics analysis also revealed the presence of helix-turn-helix and basic amino acid residues at the N-terminal nucleic acid-binding domain of CPs and indicated their importance during interactions with viral genomes (T. Ranjan, unpublished data). The thoroughly characterized packaging signals, viz., assembly sequence, tRNA-like sequence, hairpin-like structures, and some untranslated regions at the 5’- or 3’-end are recognized by CPs to discriminate viral RNA genomes from cellular RNAs (Choi and Rao, 2000; Choi et al., 2002; Bink et al., 2003; Choi and Rao, 2003). Selective packaging of the genomic RNA (gRNA) is mediated by high-affinity binding between the CP and the packaging signal (Figure 2A) (Wang et al., 1999; Choi and Rao, 2000; Choi et al., 2002; Choi and Rao, 2003; Kwon et al., 2005; Wang et al., 2005). Binding of one molecule of CP to the packaging signal changes the conformation of the CP from weak CP–CP interactions to strong interactions in a processive manner (Yang et al., 2017; Comas-Garcia, 2019). In almost all plant RNA viruses, the event of replication is functionally coupled with genome translocation, packaging, and virion assembly (Malik et al., 2005; Annamalai and Rao, 2006). A physical interaction with a virus-encoded replicase (p2a) expedites the CP to smoothly nucleate over the RNA genome and further increases the packaging specificity in an energy-dependent step (Figure 2B) (Annamalai and Rao, 2005; Annamalai and Rao, 2006; Annamalai et al., 2008; Chaturvedi and Rao, 2014; Comas-Garcia, 2019). Intriguingly, an interaction between host-encoded HSP70 and CP is critical during genome packaging and virion encapsidation in few plant RNA viruses, indicating variations within the type I genome packaging apparatus (Vriend et al., 1986; Alzhanova et al., 2001; Verchot, 2012; Gorovits et al., 2013). The disruption of such physical interactions leads to the packaging of cellular RNAs along with the viral RNA genome in a mature virion particle (Weiss et al., 1994; Wu and Shaw, 1998; Annamalai and Rao, 2005). Similarly, the inactivation of the ATPase domain of CPs employing the RNAi approach further inhibits the entire process of RNA genome packaging (Rakitina et al., 2005; Ranjan et al., 2021; Kumar et al., 2022; Mohanty et al., 2023). This confirms that genome packaging and assembly in a plant RNA virus is an ATP-dependent process (Figure 2B) (Ranjan et al., 2021; Kumar et al., 2022; Mohanty et al., 2023).

Intriguingly, the superimposed structure of the docked CP–ATP complex showed that each monomer has an active site at the interface (Ranjan et al., 2021; Kumar et al., 2022; Mohanty et al., 2023). Binding of ATP molecules to the ATPase domain of CPs brings conformational changes in the tertiary structure of proteins (Figure 2C), and furthermore, their hydrolysis favors the nucleation of CPs onto viral RNA genomes (Figure 2D). ATP hydrolysis also promotes oligomerization (monomer-to-trimer-to-pentamer) of CPs into a large capsomere-like complex structure and ultimately packages RNA genomes into mature virions (Figure 2E) (Kumari et al., 2020; Ranjan et al., 2021; Kumar et al., 2022; Mohanty et al., 2023).

## 4 Strategies to combat plant RNA viruses by targeting crucial steps of genome packaging

**FIGURE 3:**
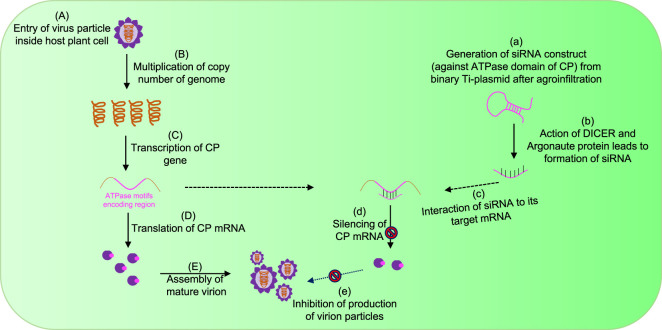
Strategy for controlling plant viruses by targeting genome packaging events during their life cycle. **(A)** Plant virus enters inside the host cytoplasm with the help of a vector. **(B)** After uncoating the genome, the virus increases its copy number by replication, and further transcription **(C)** and translation **(D)** events enable the production of CP ATPase. **(E)** Ultimately, CP encapsidates the RNA genome with the input of ATP molecules and forms mature virion particles. The introduction of exogenous siRNA constructs specific to ATPase domains suppresses the expression of CP mRNAs **(a–d)** and ultimately inhibits the production of functional virions **(e)**.

After entry into the host cytoplasm with the assistance of a vector (Figure 3A), viruses first increase their copy number of genomes via the replication process (Figure 3B). Furthermore, transcription (Figure 3C) and translation (Figure 3D) produce CPs, which ultimately help in the encapsidation of multiplied genomes into virion particles (Figure 3E). Targeting genome packaging and virus assembly processes could be one of the efficient approaches toward the development of virus resistance in plants (Ranjan et al., 2021; Kumar et al., 2022; Mohanty et al., 2023) (Figures 3a–e). One of the most effective approaches to build plant resistance has been to introduce a part of the viral gene into the plant either to induce RNA silencing against viral RNAs or to express intact or modified viral proteins or RNAs that disturb the viral infection cycle (Goldbach et al., 2003). The RNA interference (RNAi) technology has emerged as a potential tool to target virus assembly for developing resistant crops (Lodge et al., 1993; Goldbach et al., 2003) (Figure 3). RNA silencing, a conserved regulatory mechanism of gene expression in eukaryotes, is triggered by dsRNA-provoking gene silencing through sequence-specific degradation of complementary mRNA transcripts (post-transcriptional gene silencing) (Waterhouse et al., 2001). The degradation of these target RNAs occurs in a sequence-specific manner via the formation of double-stranded RNAs, which further processed into small interfering RNAs (siRNAs) by the Dicer-like (DCL) proteins and the RNA-induced silencing complex (RISC) (Lodge et al., 1993; Waterhouse et al., 2001; Goldbach et al., 2003) (Figures 3a–c). Since it is well-acknowledged that the CP is essential for plant RNA virus genome packaging (Duggal and Hall, 1993; Hacker, 1995; Kumari et al., 2020; Ranjan et al., 2021; Kumar et al., 2022; Mohanty et al., 2023), in our previous studies, an attempt was made to explore the conserved functional motifs situated across a polypeptide chain of CPs for developing strategies toward virus resistance in plants (Kumari et al., 2020; Ranjan et al., 2021; Kumar et al., 2022; Mohanty et al., 2023) (Figures 3d–e).

**FIGURE 4:**
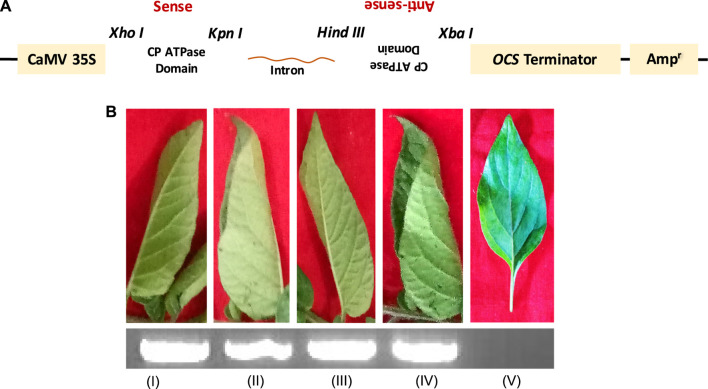
Genome packaging is an ATP-dependent process in plant viruses. **(A)** Schematic representation of the generation of siRNA construct specific to the ATPase domain of CP. **(B)** Symptoms observed in the tertiary leaves of the infected potato plants after 2 weeks of PLRV inoculation. (I) PLRV-infected control without agroinfiltration; (II) agroinfiltrated with the empty vector pART27; (III) agroinfiltrated with the plasmid containing only the antisense sequence (pART27–antisense CP_ATPase domain); (IV) agroinfiltrated with the plasmid containing only the sense sequence (pART27–sense CP_ATPase domain); and (V) agroinfiltrated with the pART27–CP_ATPase siRNA construct. The detection of the PLRV RNA using RT-PCR (inset; agarose gel). For details, refer to Kumar et al., 2022.

In our previous study, the efficiency of siRNA constructs of the CP_ATPase domain to silence and inhibit virus assembly was assessed in detail (Kumar et al., 2022). To further validate this efficient methodology, siRNAs designed against ATPase folds (comprising all motifs) of the CP were agroinfiltrated into virus-infected plants. Figure 4A depicts the details of the generation of siRNA constructs against the ATPase domain of CPs (Figure 4A). Agroinfiltrated plants did not show any symptoms of a virus infection. The suppression of viral infection could be attributed to the reduced expression of CPs due to its silencing by siRNA constructs (Figures 3, 4) (Kumar et al., 2022). To understand whether the knockdown of CPs, which possess a classical ATPase domain, can disrupt RNA virus genome packaging, we agroinfiltrated the pART27–CP_ATPase domain–siRNA constructs into potato plants (Kumar et al., 2022). Control plants (naturally virus-infected plants) (Figure 4B; lane I) and those agroinfiltrated with the empty vector (Figure 4B; lane II) showed rolling symptoms of potato leafroll virus (PLRV) infection in the upper leaves (Kumar et al., 2022). Intriguingly, the agroinfiltration of plantlets individually with sense (pART27–sense CP_ATPase) and antisense (pART27–antisense CP_ATPase) constructs of the CP_ATPase domain also displayed symptoms of leaf rolling in the upper leaves (Figure 4B; lane III and IV, respectively). This is obvious because these constructs were not able to form dsRNAs after agroinfiltration and Dicer cannot recognize them anymore. Plants agroinfiltrated with the siRNA construct of CP_ATPase (pART27–CP_ATPase) showed no symptoms of a viral infection (Figure 4B; lane V). These results demonstrated that an siRNA construct, specific to the ATPase domain (comprising all critical motifs) of CPs, leads to the suppression of viral genome packaging and assembly in plants (Kumar et al., 2022).

Our study showed that the transient expression of CP constructs specifically and efficiently inhibits genome packaging of plant RNA viruses (Kumar et al., 2022). Interestingly, CP mRNAs were found to be very high in the tertiary leaflet of control plants (natural infection of virus) and agroinfiltrated plants with an empty vector without any construct, sense construct, and antisense construct as well (Figure 4B I–IV; lower inset), whereas mRNAs of CPs were not even found in the new leaflets of agroinfiltrated plants with CP siRNAs targeted against ATPase folds (Figure 4B V; lower inset) (Kumar et al., 2022). These findings support the previous conjecture and strongly suggest a direct role of CP_ATPase in genome recognition and its packaging, the suppression of which may result in immature, non-functional, and genome-deficient viral particle production (Callaway et al., 2001; Rakitina et al., 2005; Hesketh et al., 2015).

## 5 Variations among type I genome packaging systems

Albeit the differences in the basic architecture and hosts of plant viruses, the process of assembly is coordinated by specific interactions of the nucleic acid-binding domain of capsid proteins with the viral genomic RNA followed by their oligomerization and nucleation in the genome. Linear polypeptide chains of CPs of type IA viruses encrypt a classical novel ATPase domain (Ranjan et al., 2021; Mohanty et al., 2023). On the other hand, CPs encoded by viruses in the type IB packaging and type IC packaging apparatus lack the ATPase domain and represent a variation within type I genome packaging (Ranjan et al., 2021). Our recent bioinformatics analysis revealed the relatedness of the type IB to type IC genome packaging system and the very early evolution of the type IA system. Interestingly, viruses from the type IA packaging system clustered into a separate clade and seemed to be diverged early from the rest of the two genome packaging systems during the evolution time (Kumari et al., 2020; Ranjan et al., 2021; Kumar et al., 2022; Mohanty et al., 2023). This represents a typical example of divergent evolution of the primordial viral genome packaging apparatus, and perhaps it could be the reminiscent genome packaging system encoded by the last universal common ancestor (LUCA). It seems that CPs of plant RNA viruses appear to derive from a common ancestor, regardless of their host (Ranjan et al., 2021; Mohanty et al., 2023). It could also represent a new variation and the adaptive evolution of the ancient viral genome packaging apparatus that likely existed in the LUCA (Burroughs et al., 2007; Chelikani et al., 2014b; Iyer et al., 2004; Mohanty et al., 2023; Ranjan et al., 2021).

## 6 Conclusion

RNA viruses cause serious diseases in crops, leading to significant reductions in annual yields worldwide. Innovative and state-of-the-art strategies are very much required to control/combat with plant viruses in order to meet the demands of the population growth worldwide (Hill et al., 2021; Ye et al., 2021). Among several advances in viral control measures, aiming for the steps of the viral assembly process has the potential to save crops from such destructive diseases (Burroughs et al., 2007; Dai et al., 2021; Kumar et al., 2022). Understanding the mechanism of genome packaging and assembly would be helpful in developing novel approaches to control plant viruses and ultimately to meet the demands of a growing world population (Kumar et al., 2020; Ranjan et al., 2021; Kumar et al., 2022; Mohanty et al., 2023). Deciphering the detailed machinery of ATP binding and catalysis by the ATPase fold would be helpful in designing novel inhibitors, which may hinder the entire process of genome packaging and virus assembly (Twarock and Stockley, 2019). Information collected from the limited available literature worldwide on Polerovirus and Potexvirus as model systems has provided novel insights into understanding the mechanism of ATP-dependent genome packaging and virus assembly. The extension of similar studies to other plant RNA viruses would be helpful in determining whether ATP-dependent genome packaging is universally conserved among different RNA viruses infecting plants. Still, important questions regarding the mechanisms regulating the process of genome packaging and assembly of uniformly sized virions remain unanswered and a challenge for future investigation.
